# The Risk Factors for Progression to Chronic Pancreatitis in Patients with Past-History of Acute Pancreatitis: A Retrospective Analysis Based on Mechanistic Definition

**DOI:** 10.3390/jcm11082209

**Published:** 2022-04-15

**Authors:** Akira Yamamiya, Keiichi Tominaga, Koki Hoshi, Kazunori Nagashima, Takahito Minaguchi, Yasuo Haruyama, Atsushi Irisawa

**Affiliations:** 1 Department of Gastroenterology, School of Medicine, Dokkyo Medical University, 880 Kitakobayashi Mibu, Tochigi 321-0293, Japan; tominaga@dokkyomed.ac.jp (K.T.); hoshi@dokkyomed.ac.jp (K.H.); n-kazu@dokkyomed.ac.jp (K.N.); takahito@dokkyomed.ac.jp (T.M.); irisawa@dokkyomed.ac.jp (A.I.); 2 Integrated Research Faculty for Advanced Medical Science, School of Medicine, Dokkyo Medical University, 880 Kitakobayashi Mibu, Tochigi 321-0293, Japan; yasuo-h@dokkyomed.ac.jp

**Keywords:** chronic pancreatitis, acute pancreatitis, mechanistic definition, Alcohol Use Disorders Identification Test-Concise

## Abstract

Background: According to the mechanistic definition, the history of acute pancreatitis (AP) is a risk factor for chronic pancreatitis (CP). However, the etiology and severity of previous AP involved in the progression to CP have not been clarified. Here, we investigated risk factors for the progression to CP in patients with past-history of AP. Methods: Sixty-four patients with AP who were followed-up for at least two years at our institution between April 2009 and March 2017 were enrolled. The multivariate analysis was performed based on the risk factors extracted by univariate analysis. Results: Among the 64 patients, 13 patients (20.3%) progressed to CP (PCP group), while 48 did not (non-PCP group). Regarding the etiology of AP, rate of alcohol AP was significantly higher in the PCP group (76.9% vs. 33.3%, *p* = 0.003). In univariate analysis, smoking, number of previous AP, and alcohol consumption and drinking habits (Alcohol Use Disorders Identification Test-Concise; AUDIT-C) were identified as factors associated with progression to CP. Furthermore, multivariate analysis showed that AUDIT-C ≥ 6 points (male) and 4 points (female) after AP was a significant risk factor for CP (*p* = 0.003). Conclusions: Our results indicated that AUDIT-C ≥ 6 points (male) and 4 points (female) after AP was a risk factor in the process of progression to CP in patients with past-history of AP.

## 1. Introduction

Chronic pancreatitis (CP) is an irreversible and progressive inflammation of the pancreas. CP is characterized by extensive pancreatic gland fibrosis due to persistent and recurrent inflammation, which eventually leads to pancreatic exocrine and endocrine secretion disorders [1,2]. Based on the recently proposed mechanistic definition [3], CP was defined as “pathologic fibro-inflammatory syndrome of the pancreas in individuals with genetic, environmental, and/or other risk factors who develop persistent pathologic responses to parenchymal injury or stress”. Its incidence is increasing globally [4,5,6]. CP is associated with a high incidence of pancreatic cancer, and early diagnosis and intervention can aid in preventing disease progression [7,8,9,10,11]. In 2009, the diagnostic criteria for early chronic pancreatitis (DCECP2009) were proposed by the Japan Pancreas Society [12].

Ten years after DCECP2009 was established, the early CP (ECP) criteria were revised in 2019 (DCECP2019) [5,13]. In this revision, the EUS diagnostic criteria were simplified to four items from the previous seven items, which has improved the interobserver reliability [14,15], and is expected to improve the diagnostic specificity. In addition, a mechanistic definition was incorporated as a concept of CP, and the risk factors for pancreatitis other than alcohol were also added to the early CP (ECP) diagnostic criteria. The diagnostic criteria for ECP was revised in terms of risk factors. In this revision, based on accumulated data and expert opinion, three of five clinical features, including recurrent abdominal pain or back pain, blood and urinary pancreatic enzyme abnormalities, pancreatic exocrine disorder, continuous heavy drinking of alcohol equivalent to or more than 60 g/day of pure ethanol (EtOH 60 g/day), and pancreatitis-related susceptibility genes and past-history of acute pancreatitis (AP), were revised.

Past-history of AP was added to the list of clinical features because AP and recurrent pancreatitis have been shown to be important in leading to ECP in the mechanistic definition of CP. In a prospective observational study of ECP conducted in Japan, past-history of AP was reported to be significantly associated with progression to probable CP [16]. However, not all subjects with past-history of AP progressed to CP, and the reason for this difference is not clear. There are several reports that alcohol and smoking are involved in recurrent pancreatitis (RAP) and the progression of AP to CP [17,18,19,20,21,22]. These reports examined that heavy drinkers and smokers tended to progress to RAP/CP compared to non-heavy drinkers and smokers. However, their specific consumption and habits have not been clarified. The Alcohol Use Disorders Identification Test-Concise (AUDIT-C) is an objective evaluation score for alcohol consumption and drinking habits, and is widely used as an evaluation of alcohol dependence. Therefore, in this study, we investigated the risk factors for the progression to CP in patients with past-history of AP by using AUDIT-C, which can objectively assess alcohol consumption and habits.

## 2. Methods

### 2.1. Study Design

**Figure 1:**
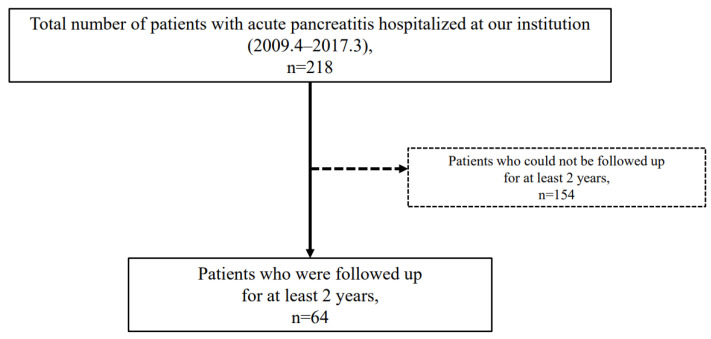
Flow diagram of this study.

This study was conducted at Dokkyo Medical University and was approved by our institutional Medical Ethics Committee (R-39-1J). The study was registered with the University Hospital Medical Information Network (UMIN) Clinical Trials Registry (000051014). A means to opt out was provided instead of omitting informed consent, which enabled research subjects to be notified and allowed publication of research information on our website. A total of 218 patients were hospitalized with AP at our institution between April 2009 and March 2017. Among these, 64 patients who were followed-up for at least 2-years were analyzed (Figure 1). AP patients with relapse of CP were excluded from this study. In addition, these 64 patients were confirmed not to have CP by CT, MRCP, or EUS at the first onset of AP. To identify the risk factors for CP progression from AP, we performed univariate analysis. Multivariate analysis was subsequently performed based on the extracted risk factors. The EtOH was analyzed for each 20 g unit, and the presence or absence of smoking, another risk factor for CP, was also combined in the analysis.

The primary endpoints were to examine the risk factor in the process of progression to CP in patients with past-history of AP. The secondary endpoint was to evaluate the rate of progression from AP to CP.

### 2.2. Severity of AP

The Japanese criteria for the assessment of AP severity developed by the Research Committee of Intractable Diseases of the Pancreas (Ministry of Health, Labor and Welfare) were used for evaluating the prognostic factors and CE-CT grading for AP (Supplementary Table S1) [23]. Prognostic factors consisted of the following nine items: (1) base excess (BE) ≤ 2–3 mEq/L or shock (systolic blood pressure < 80 mmHg), (2) PaO_2_ ≤ 60 mmHg (room air) or requiring respirator management, (3) blood urea nitrogen ≥ 40 mg/dL (or creatinine ≥ 2.0 mg/dL) or oliguria after fluid replacement, (4) lactic dehydrogenase (LDH) twice the upper limit of normal, (5) platelet count ≤ 100,000/mm^3^, (6) serum calcium ≤ 7.5 mg/dL, (7) C-reactive protein ≥ 15 mg/dL, (8) number of positive measures in the systematic inflammatory response syndrome (SIRS) criteria ≥ 3, and (9) age ≥ 70 years. Patients who satisfy more than three of the above nine items are assessed as having severe AP. The CE-CT grade is a classification for severity assessment made by the combination of two factors: the degree of extra-pancreatic progression of inflammation and the extent of LEPP, and cases of grade 2 or above are considered severe.

In addition, all cases were also evaluated for AP severity using the revised Atlanta classification [24]. Mild acute pancreatitis is characterized by the absence of organ failure and the absence of local or systemic complications. Moderately severe acute pancreatitis is characterized by the presence of transient organ failure or local or systemic complications in the absence of persistent organ failure. Severe acute pancreatitis is characterized by persistent organ failure.

### 2.3. Diagnostic Criteria for CP 2019

Clinical findings of chronic pancreatitis are: (1) characteristic imaging findings, (2) characteristic histological findings, (3) repeated upper abdominal pain or back pain, (4) abnormal pancreatic enzyme levels in the serum or urine, (5) abnormal pancreatic exocrine function, (6) continuous heavy drinking of alcohol equivalent to or more than 60 g/day of pure ethanol (EtOH 60 g/day) or pancreatitis-related susceptibility genes, and (7) past-history of acute pancreatitis. Definite CP is defined as a case that satisfies definite findings in (1) or (2), or probable findings in (1) or (2) and two or more features of (3), (4), or (5). Probable CP is defined as the cases that satisfy probable findings in (1) or (2). ECP is defined as a case in which two or more features from (3) to (7) are satisfied and the image findings of early CP are confirmed. Cases that have only finding (1) or (2) and show the findings of early CP are diagnosed as probable early CP (Supplementary Table S2) [5,13].

### 2.4. Assesment of Alcohol Consumption and Drinking Habits

Alcohol consumption and drinking habits were assessed using a short form of the AUDIT-C, a modified version of the 10-question Alcohol Use Disorders Identification Test (AUDIT) developed by the World Health Organization (Supplementary Table S3) [25,26]. This test is a brief, self-reported alcohol screening test effective for assessing unhealthy alcohol use. This instrument is a 3-item survey with a total score ranging from 0 to 12 points. A score of 3 or more points on the AUDIT-C could indicate people who are at-risk drinkers or have alcohol use disorders. A score of 6 points for male and 4 points for female or more for each is classified as the ‘High-risk drinking’ group, taking into account the differences in alcohol sensitivity between males and females. In general, the alcohol abuse is directly proportional to the highest score on the test [27,28,29].

### 2.5. Statistical Analysis

Continuous variables were reported as means ± standard deviation (SD), whereas categorical data were expressed as frequencies. The Mann–Whitney U test was used to compare the differences in continuous variables, and the Pearson χ^2^ test or Fisher exact test was used to compare categorical variables between the two groups. Univariable and multivariable logistic regression were used to determine risk factors. *p* < 0.05 was considered statistically significant. All statistical analyses were performed using SPSS software version 27.0 (International Business Machines Co., Tokyo, Japan).

## 3. Results

### 3.1. Overall Patient Characteristics

Overall patient characteristics are presented in Table 1. Of the 64 patients with a mean age of 58 years (±16 years), 46 (72%) were male and 18 (28%) had a history of AP. The most frequent etiology was alcohol (26 cases, 41%). In addition, 12 cases (19%) were gallstone-related, three cases (5%) were drug-induced, three cases (5%) had dyslipidemia, and one case (2%) had pancreaticobiliary malfunction. The AP severity at the time of onset according to the Japanese criteria was severe in 25 cases and mild in 39 cases. According to the revised Atlanta classification, the AP severity was mild in 39 cases, moderately severe in 16 cases, and severe in 9 cases. The mean observation period was 59 months, and the final diagnoses during the observation period were normal in 48 cases (75%), progression to possible ECP in 2 cases (3.1%), progression to definite diagnosis of ECP in 1 case (1.6%), and progression to definite CP in 13 cases (20.3%).

### 3.2. Analysis of Risk Factors for CP Progression from AP

#### 3.2.1. Comparison of Patient Characteristics between Progression and Non-Progression to CP

Among 64 patients with AP who were followed-up for at least 2 years, 13 patients progressed to CP (PCP group), while 48 did not (non-PCP group, excluding ECP). Patient characteristics between the two groups were compared, and the results are presented in Table 2. The mean observation period was similar between the 2 groups, with 61 months in the PCP group and 59 months in the non-PCP group (*p* = 0.933). No significant differences between the two groups were observed for age, sex, AP severity at time of onset and within 48 h, prognostic factor score at time of onset and within 48 h, and CE-CT grade at time of onset. The revised Atlanta classification and APACHE-II score were not significantly different between the two groups. For the etiology of AP, 76.9% (10/13) in the PCP group and 33.3% (16/48) in the non-PCP group had alcohol pancreatitis, and the percentage was significantly higher in the PCP group (*p* = 0.003). There was no significant difference in the prevalence of chronic kidney disease, diabetes mellitus (DM), heart disease, dyslipidemia, and hypertriglyceridemia between the two groups.

#### 3.2.2. Analysis of Clinical Findings Related to Diagnostic Criteria for CP 2019

The results of clinical findings related to CP are presented in Table 3. The mean EtOH intake was significantly higher in the PCP group (60 g/day) compared with the non-PCP group (33 g/day) (*p* = 0.005). The percentage of patients with EtOH > 60 g/day was significantly higher in the PCP group (46.2%, 6/13) compared with the non-PCP group (6.3%, 3/48) (*p* = 0.021). AUDIT-C before AP was significantly higher in the PCP group (8.9 points) compared with the non-PCP group (4.4 points) (*p* = 0.006). AUDIT-C after AP was significantly higher in the PCP group (8.2 points) compared with the non-PCP group (0.9 points) (*p* < 0.001). As for smoking, the Brinkman index was similar between both groups (310 vs. 259, *p* = 0.147). The percentage of continued smoking was similar between both groups (61.5% vs. 50%, *p* = 0.326). In the PCP group, 92.3% (12/13) showed a pancreatic stone on imaging examination and 69.2% (9/13) showed a dilated pancreatic duct. The number of past AP occurrences was significantly higher in the PCP group (1/2/3/4/5 times = 2/9/1/1/0) compared with the non-PCP group (1/2/3/4/5 times = 38/6/2/2/1) (*p* = 0.025).

#### 3.2.3. Univariate and Multivariate Analysis of Risk Factors for CP Progression in Patients with Past-History of AP

The results of risk factors for progression to CP in patients with past-history of AP are presented in Table 4. In univariate analysis, Brinkman index ≥ 400, number of past AP occurrences, and AUDIT-C ≥ 6 points (male) and 4 points (female) before/after AP were identified as factors associated with progression to CP. Furthermore, multivariate analysis showed that only AUDIT-C ≥ 6 points (male) and 4 points (female) after AP was a significant risk factor for progression to CP (*p* = 0.003).

## 4. Discussion

Recently, Whitcomb et al. proposed a mechanistic definition that incorporates the concept of ECP [3]. This is a mechanistic idea that individuals with risk factors develop ECP by repeating AP and then progress to established CP and end-stage CP. In this progression, ECP is considered to be a condition with residual pancreatic function and reversible characteristics. The reversibility of fibrosis and functional recovery of the human body has been reported in other organs, including improvement of liver fibrosis after viral elimination in viral cirrhosis [30,31,32,33]. The improvement of exocrine and endocrine disturbances during the healing process of autoimmune pancreatitis and AP is another example of the reversibility of mechanical disturbances [34]. Consequently, early diagnosis of CP requires an understanding and mechanistic definition of CP, with close attention paid to the pathological process leading to CP, as opposed to focusing only on the pathological and clinical states of progressed CP. From this perspective, the DCECP2019 added pancreatitis-related susceptibility genes and past-history of AP as risk factors for CP to the clinical diagnosis items.

According to the mechanistic definition, AP and recurrent pancreatitis are important in progression to CP [3]. In a nationwide survey of ECP in Japan, cases with definite/probable CP were more likely to be male (4.8% vs. 1.3%), to have a history of heavy alcohol consumption (72.0% vs. 45.8%), to have a history of smoking (69.6% vs. 41.0%), to have DM (42.3% vs. 19.3%), and to have a history of AP (50.4% vs. 22.1%) compared with cases with ECP [35]. The male to female ratio of ECP was 1.4:1, the mean age was 61.9 years, the mean age of onset was 56.7 years, and the etiology was alcohol and idiopathic (44% for each). Thus, ECP tended to have a higher proportion of men, more idiopathic cases, and an older mean age of onset. However, there were discrepancies in the patient profile and a reversal of the mean age of onset between the confirmed cases of ECP and CP. This discrepancy disappeared when the study was limited to ECP patients with past-history of AP. In addition, in a prospective observational study of ECP, 4 of 83 patients (4.8%) with ECP who were followed-up for 2 years progressed to definite CP [16]. Comparison between patients show that alcohol, a history of smoking, and a history of AP were associated with progression to definite CP. Lankisch et al. also conducted a long-term follow-up study of AP and reported a progression rate to CP of 16% over 20 years [21]. They added that the only progression from AP to CP was alcohol CP. Ammann et al. also reported that up to 78% of patients with alcoholic AP progressed to CP over 16 years [36,37]. However, these reports only state that alcoholic AP is a risk factor for progression to RAP and CP, and a detailed examination of alcohol consumption and drinking habits has not been conducted to date [36]. Furthermore, there are few studies on the differences in AP severity at the time of onset and etiology in AP patients and the subsequent progression to CP.

In the present study, we investigated the progression to CP in patients with past-history of AP and compared the differences in AP severity at the time of onset and the etiology between those that progressed to CP and those that did not. In fact, among 64 patients with past-history of AP, 1 case (2%) developed ECP and 13 cases (20%) developed CP. Based on the mechanistic definition, ECP progresses to established CP over a period of months due to injury or stress [3]. In addition, it progresses to the end-stage CP in months to years. With regards to the mechanism of the progression from AP to CP, Kloppel and Maillet suggested that RAP might lead to CP (necrotic-fibrosis sequence hypothesis) [38]. Thus, it is natural to assume that the 13 patients who progressed to CP in this study also progressed through ECP, based on the mechanistic definition. Besides, the one case that progressed to ECP may progress to CP depending on the future course of the disease. This supports the idea that patients with past-history of AP may progress to ECP/CP, and suggested that it was appropriate to incorporate the concept of the mechanistic definition into the clinical diagnosis criteria for CP. Furthermore, in this study, smoking, number of past AP occurrences, and AUDIT-C ≥ 6 points (male) and 4 points (female) after/before AP were extracted as risk factors for progression to CP by univariate analysis, similar to a previous report by Masamune et al. [35]. In addition, our multivariate analysis showed that AUDIT-C ≥ 6 points (male) and 4 points (female) after AP was a significant risk factor in the process of progression to CP. The heavy drinking history criterion was changed from EtOH > 80 to 60 g/day in this revision of the diagnostic criteria because EtOH > 60 g is defined as a habitual drinker in the diagnostic criteria for alcoholic liver disease. Moreover, a case-control study in Japan showed that more alcohol consumption than 20–40 g/day was a risk factor for CP regardless of past-history of AP [39]. As such, it is generally recognized as a risk factor for alcohol AP and progression to CP. For example, Ammann et al. stated that alcohol consumption in excess of EtOH 80 g/day for five years would progress to CP [40]. Layer et al. set a lower upper limit of 50 g/day for alcohol consumption, but did not specify the duration of alcohol abuse before developing CP [41]. Up to the present day, no established upper limits for the amount and duration of alcohol consumption that progresses to CP have been established [42]. Our study was the first to report the AUDIT-C ≥ 6 points (male) and 4 points (female) after AP, which was extracted as a risk factor in the process of progression to CP with past-history of AP. This result suggested that not only alcohol consumption but also drinking habits may be involved in the progression of CP. Furthermore, this study was the first report to objectively assess alcohol consumption and habits as risk factors for CP. The advantage of using the AUDIT-C to identify risk factors for CP progression is that this score includes gender differences. Women are generally smaller in size, liver, and muscle mass and more susceptible to alcohol than men, and may develop CP with smaller amounts of alcohol consumption [18].

On the other hand, AUDIT-C before AP and number of past AP occurrences were extracted as risk factors in the univariate analysis. Repeated APs may lower the threshold for alcohol-induced pancreatic damage. Therefore, even if alcohol consumption is low, AP may be more likely to develop. Thus, repeated AP due to alcohol intake may lead to CP. From these perspectives, for patients with alcoholic AP, a stricter definition of heavy alcohol consumption and drinking habits may be required to prevent the progression to CP. In addition, it has been pointed out that smoking is a risk factor for developing CP [19,20,43,44]. Based on the univariate analysis in our study, smoking may contribute to CP development in patients with a history of AP. The combination of smoking and alcohol consumption was expected to be a high-risk factor for CP progression in patients with past-history of AP. Ahmad et al. reported that the cumulative risk of CP progression was increased to 30% in smokers and heavy drinkers compared to non-smokers [20]. Yadav et al. also found an increased risk of CP among heavy drinkers with smoking (OR, 4.69; 95% CI, 2.76–7.97), as well as an increased risk among heavy smokers with drinking (OR, 2.35; 95% CI, 0.71–7.78), compared to heavy smokers with no-drinking (OR, 8.07; 95% CI, 4.97–13.1) [45]. However, in this present study, this combination was not extracted as a significant factor because the number of patients with both factors was small. Smoking-induced acinar cell injury and chronic vascular damage have been shown to induce AP/CP [46,47,48,49,50], and smoking as a risk factor for progression to CP also warrants further study.

There were several limitations in our study, such as the single-center and retrospective design, and the number of cases was small. In addition, there was no predefined protocol for the follow-up. Therefore, some of the cases had a short follow-up period. However, all patients were followed-up for at least two years after AP onset, and 20% progressed to CP. This suggested that patients with past-history of AP might progress to CP after a period of years, as shown in the mechanistic definition. In contrast, there was one case that only progressed to ECP, but it was not included in the analysis because ECP does not always progress to CP. Due to the retrospective design, we could not capture all cases that progressed to ECP. Future studies should prospectively analyze the progression to ECP in patients with past-history of AP.

In conclusion, our results suggested that it was appropriate to incorporate the concept of mechanistic definition into the clinical criteria for CP, as patients with past-history of AP might progress to CP at a certain rate. Moreover, AUDIT-C ≥ 6 points (male) and 4 points (female) after AP was extracted as a risk factor for the process of progression to CP in patients with a history of AP. Therefore, a stricter definition of heavy alcohol consumption and drinking habits including gender differences may be useful in preventing the progression to CP.

## Figures and Tables

**Table 1:** Overall patient characteristics.

Age, mean ± SD (range), years	58 ± 16 (18–89)
Sex, male/female, *n*	46/18
Observation period, mean ± SD (range), months	59 ± 33 (24–158)
Etiology, *n* (%)	
Alcohol	26 (41)
Stone in biliary system	12 (19)
Drug-induced	3 (5)
Dyslipidemia	3 (5)
Pancreaticobiliary maljunction	1 (2)
Severity of AP at the onset according to the Japanese criteria: Mild/Severe, *n*	39/25
Prognostic factor score at the onset, mean ± SD (range), points	1 ± 1.4 (0–7)
CE-CT Grade at the onset: Grade 1/2/3, *n*	40/20/4
Severity of AP at the onset according to revised Atlanta classification: Mild/Moderate, Severe/Severe, *n*	39/16/9
Number of past-history of AP: 1/2/3/4/5, times	43/15/3/2/1
APACHE II score, mean ± SD (range), points	3.5 ± 2.7 (2–15)
History of cardiovascular disease, *n* (%)	10 (16)
History of chronic kidney disease, *n* (%)	6 (9)
History of diabetes mellitus, *n* (%)	13 (20)
History of dyslipidemia, *n* (%)	23 (36)
History of hypertriglyceridemia, *n* (%)	12 (19)
Amount of continuous drinking of alcohol, EtOH(g), mean ± SD (range)	39 ± 57 (0–265)
AUDIT-C before AP	5.3 ± 4.9 (0–12)
AUDIT-C after AP	2.4 ± 4.4 (0–12)
Progression to CP, *n* (%)	13 (20)
Progression to ECP, *n* (%)	1 (2)

SD, standard deviation; AP, acute pancreatitis; AUDIT-C, Alcohol Use Disorders Identification Test-Concise; CE-CT, contrast-enhanced computed tomography; APACHE II, Acute Physiology and Chronic Health Evaluation II; CP, chronic pancreatitis; ECP, early chronic pancreatitis.

**Table 2:** Comparison of patient characteristics between PCP and non-PCP groups.

	PCP Group (*n* = 13)	Non-PCP Group (*n* = 48)	*p*-Value
Age, mean ± SD (range), years	52 ± 15 (28–79)	60 ± 14 (18–89)	0.063
Observation period, mean ± SD (range), months	61 ± 25 (24–158)	59 ± 21 (25–135)	0.933
Sex, male/female, *n*	12/1	31/17	0.066
Etiology due to alcohol, *n* (%)	10 (77)	16 (33)	0.003
Severity of AP at the onset according to the Japanese criteria: Mild/Severe, *n*	6/7	18/30	0.609
Prognostic factor score at the onset, mean ± SD (range), points	1 ± 2.1 (0–7)	1 ± 0.9 (0–5)	0.265
CE-CT grade at the onset: Grade 1/2/3, *n*	7/5/1	33/14/1	0.481
Severity of AP at the onset according to revised Atlanta classification: Mild/Moderate, Severe/Severe, *n*	7/4/2	32/12/4	0.598
APACHE II score, mean ± SD (range), points	4 ± 3.8 (2–15)	3.4 ± 2.1 (2–12)	0.660
History of cardiovascular disease, *n* (%)	1 (8)	8 (17)	0.378
History of chronic kidney disease, *n* (%)	1 (8)	4 (8)	0.816
History of diabetes mellitus, *n* (%)	3 (23)	9 (19)	0.133
History of dyslipidemia, *n* (%)	5 (39)	15 (31)	0.157
History of hypertriglyceridemia, *n* (%)	2 (15)	8 (16)	0.201

SD, standard deviation; AP, acute pancreatitis; CE-CT, contrast-enhanced computed tomography; APACHE II, Acute Physiology and Chronic Health Evaluation II.

**Table 3:** Analysis of clinical signs related to CP.

	PCP Group (*n* = 13)	Non-PCP Group (*n* = 48)	*p*-Value
The appearance of pancreatic stone, *n* (%)	12 (92)	0 (0)	-
The appearance of dilated pancreatic duct, *n* (%)	9 (69)	0 (0)	-
Repeated upper abdominal pain or back pain, *n* (%)	3 (23)	7 (15)	0.657
Abnormal pancreatic enzyme levels in the serum or urine, *n* (%)	3 (23)	4 (8)	0.196
Amount of continuous drinking of alcohol, EtOH (g), mean ± SD (range)	60 ± 52 (0–130)	33 ± 56 (0–265)	0.005
EtOH > 20 g/day, *n* (%)	11 (85)	18 (38)	<0.001
EtOH > 40 g/day, *n* (%)	10 (77)	17 (35)	<0.001
EtOH > 60 g/day, *n* (%)	6 (46)	3 (6)	0.021
AUDIT-C before AP	8.9 ± 3.7 (0–12)	4.4 ± 4.8 (0–12)	0.006
AUDIT-C after AP	8.2 ± 4.8 (0–12)	0.9 ± 2.8 (0–12)	<0.001
Brinkman index, mean ± SD (range)	310 ± 336 (0–968)	259 ± 451 (0–2120)	0.147
Continue smoking after AP	8 (62)	24 (50)	0.025
Number of past-history of AP: 1/2/3/4/5, times	2/9/1/1/0	38/6/2/1/1	0.025

SD, standard deviation; AUDIT-C, Alcohol Use Disorders Identification Test-Concise; AP, acute pancreatitis.

**Table 4:** Analysis of risk factors for CP progression from AP.

		Univariate Analysis			Multivariate Analysis		
		Odd-Value	*p*-Value	95% CI	Odd-Value	*p*-Value	95% CI
Age	≥60 years	1.924	0.294	1.250–2.962			
Sex	Male	0.277	0.298	0.153–0.501			
Severity of AP at the onset according to the Japanese criteria							
Severity	Severe	0.436	0.67	0.286–0.667			
Prognostic factor score at the onset	≥3 points	0.280	0.745	0.169–0.464			
CE-CT grade	≥2	0.418	0.472	0.273–0.638			
Continue Smoking after AP	Yes	1.597	0.246	0.185–1.556			
Brinkman index	≥400	1.484	0.128	0.781–2.817	2.329	0.456	0.252–21.505
History of diabetes mellitus	Yes	1.365	0.317	0.167–1.742			
History of dyslipidemia	Yes	1.251	0.426	0.145–1.963			
History of hypertriglyceridemia	Yes	1.523	0.278	0.103–1.865			
Number of past-history of AP	≥2 times	1.965	0.196	1.272–3.034	1.024	0.369	0.868–2.096
Alcohol consumption	EtOH > 20 g/day	1.103	0.208	0.022–1.540			
	EtOH > 40 g/day	1.665	0.216	0.036–3.364			
	EtOH > 60 g/day	1.456	0.205	0.026–3.754			
EtOH > 40 g/day + smoking	Yes	1.203	0.089	0.125–2.652			
AUDIT-C before AP	≥6 points (male), 4 points (female)	9.371	0.025	5.346–16.428	1.297	0.840	0.105–16.046
AUDIT-C after AP	≥6 points (male), 4 points (female)	61.229	<0.001	35.953–104.276	35.570	0.003	3.421–376.118

AP, acute pancreatitis; CE-CT, contrast-enhanced computed tomography; AUDIT-C, Alcohol Use Disorders Identification Test-Concise.

## Data Availability

No new data were created or analyzed in this study. Data sharing is not applicable to this article.

## Supplementary Materials

The following supporting information can be downloaded at: https://www.mdpi.com/article/10.3390/jcm11082209/s1, Table S1: Japanese severity scoring system for acute pancreatitis (Ministry of Health, Labour and Welfare of Japan, 2008 revision); Table S2: Clinical diagnostic criteria for chronic pancreatitis 2019; Table S3: Alcohol Use Disorders Identification Test-Concise (AUDIT-C).
