# Supplementary material for: The Risk Factors for Progression to Chronic Pancreatitis in Patients with Past-History of Acute Pancreatitis: A Retrospective Analysis Based on Mechanistic Definition
Source: J Clin Med. 2022 Apr 15;11(8):2209. doi: 10.3390/jcm11082209 (PMC9032682; doi:10.3390/jcm11082209)
Supplement: Supplementary file 1 [file jcm-11-02209-s001.zip › jcm-1606755-supplementary.pdf]

Supplementary Table S1. **Japanese severity scoring system for acute pancreatitis (Ministry of Health, Labour and Welfare of Japan, 2008 revision).**

Prognostic factors (1 point for each factor)

1. Base excess ≤ –3 mEq/L or shock (systolic blood pressure <80 mmHg)
2. P_aO_2 ≤60 mmHg (room air) or respiratory failure (requiring ventilatory management)
3. BUN ≥40 mg/dl or creatinine ≥2.0 mg/dl or oliguria (daily urine output <400 ml even after intravenous fluid resuscitation)
4. Lactate dehydrogenase ≥2 ULN
5. Platelet count ≤100 000/mm^3
6. Serum Ca ≤7.5 mg/dl
7. C-Reactive protein ≥15 mg/dl
8. No. positive measures in SIRS criteria ≥3
9. Age ≥70 y

CT grade based on contrast-enhanced CT

1 Extrapancreatic progression of inflammation	
Anterior pararenal space	0 points
Root of mesocolon	1 point
Beyond lower pole of kidney	2 points
2. Hypo-enhanced lesion of the pancreas	
The pancreas is conveniently divided into three segments (head, body, and tail)	
Localized in each segment or only surrounding the pancreas	0 points
Extends to two segments	1 point
Occupies two entire segments or more	2 points
1 + 2 = total score	
Total score = 0 or 1	Grade 1

Total score = 2	Grade 2
Total score ≥3	Grade 3
Assessment of severity	
If the prognostic factors score is ≥3 or CT grade is ≥2, the disease is graded as 'severe'.	

The systemic inflammatory response syndrome (SIRS) criteria include body temperature >38°C or <36°C, heart rate >90 b.p.m., respiratory rate >20 breaths/min or P_{aCO_2} ≤32 torr, and white blood cell counts >12 000 cells/mm^3, <4000 cells/mm^3, or >10% immature (band) forms.

BUN, blood urea nitrogen; ULN, upper limit of normal; CT, computed tomography.

Supplementary Table S2. Clinical diagnostic criteria for chronic pancreatitis 2019.

<u>**Clinical features**</u>

(1) Characteristic imaging findings

(2) Characteristic histological findings

(3) Repeated upper abdominal pain or back pain

(4) Abnormal pancreatic enzyme levels in the serum or urine

(5) Abnormal pancreatic exocrine function

(6) Continuous heavy drinking of alcohol equivalent to or more than 60 g/day of pure ethanol (EtOH 60g/day) or pancreatitis-related susceptibility genes

(7) Past-history of acute pancreatitis

<u>**Imaging findings of early chronic pancreatitis (Either a or b)**</u>

a. More than two features among the following four features of EUS findings including at least one of (1)-(2)

(1) Hyperechoic foci; non-shadowing/Stranding

(2) Lobularity [Nonhoneycombing/ honeycombing type]

(3) Hyperechoic main pancreatic duct margin

(4) Dilated side branches

b. Irregular dilatation of more than three duct branches on ERCP or MRCP findings

---

Supplementary Table S3 Alcohol Use Disorders Identification Test-Concise (AUDIT-C).

**Question 1:<br>How often did you have a drink containing alcohol in the past year?**	
**Answer**	**Points**
Never	0
Monthly or less	1
2-4 times per month	2
2-3 times per week	3
≧4 times per week	4
**Question 2:<br>How many drinks did you have on a typical day when you were drinking in the past year?**	
**Answer**	**Points**
None, I do not drink	0
1 or 2	0
3 or 4	1
5 or 6	2
7 to 9	3
10 or more	4
**Question 3:<br>How often did you have six or more drinks on one occasion in the past year?**	
**Answer**	**Points**
Never	0
Less than monthly	1
Monthly	2
Weekly	3
Daily or almost daily	4
